# Trial evaluating overall survival in epithelial ovarian cancer (eoc) patients in second remission with an autologous dendritic cell therapy targeting mucin 1

**DOI:** 10.1186/2051-1426-2-S3-P72

**Published:** 2014-11-06

**Authors:** Sharron Gargosky, Canvas Clinical Team

**Affiliations:** 1 PrimaBioMed, United States; 2 Belgium, Belarus, Bulgaria, Lithuania, Latvia, Ukraine, United States

## Rationale

Cvac is well tolerated with no serious adverse events related to Cvac treatment in CAN-003. Treatment with Cvac results in a mucin 1 specific T cell response. In the second remission patients the median PFS for Cvac was greater than 12.91 months versus a median PFS for SOC of 4.94 months (HR 0.32, p = 0.04) and in overall survival SOC groups had a median of 26.25 months, consistent with literature but CVac median is not yet reached at 30 months. Thus, based on these compelling PFS signals, Prima is now moving forward with a 210-patient study of EOC patients in second remission as compared to standard of care (SOC).

## Trial design

CANVAS is a multinational, multicenter, randomized trial of Cvac (autologous dendritic cells [DCs] pulsed with recombinant human fusion protein [mucin 1-glutathione S-transferase] coupled to oxidized polymannose) compared with standard of care as maintenance treatment in patients with EOC with no evidence of disease (NED) following second remission defined as after response to second-line platinum-based therapy.

## Eligibility

To be eligible for participation patients must have had first-line platinum-based chemotherapy with a first remission lasting for at least 6 months prior to relapse. Patients must have a second remission defined as: 1) no definitive evidence of disease detected by computed tomography (CT) or magnetic resonance imaging (MRI) of the abdomen and pelvis; 2) cancer antigen 125 (CA-125) tumor marker within normal limits OR at least a 90% reduction from pretreatment levels at the start of second-line platinum based therapy; and 3) negative physical exam (i.e., no clinical signs) following standard platinum-based second-line chemotherapy (at least 3 cycles). Patients must have a tumor that over-expresses mucin 1, as well as meet all other study eligibility criteria.

## Objectives

The primary objective is to assess the efficacy, in terms of overall survival (OS) with the secondary objectives of time to next treatment (TTNT), progression-free survival (PFS) and safety and tolerability of Cvac compared with SOC.

## Clinical centers

The trial is active in Belgium, Bulgaria, Lithuania, Latvia, Ukraine and Belarus, with other countries becoming active such as Germany. Enrolment is underway and metrics will be presented.

